# Aniracetam Ameliorates Attention Deficit Hyperactivity Disorder Behavior in Adolescent Mice

**DOI:** 10.1523/ENEURO.0578-24.2025

**Published:** 2025-03-17

**Authors:** Xiao-Li Sun, Jie Cui, Hui Bai, Wei Zhang, Wan-Jun Bai

**Affiliations:** ^1^Department of Pharmacy, The Fourth Hospital of Hebei Medical University, Hebei Key Laboratory of Clinical Pharmacy, Shijiazhuang, Hebei 050017, China; ^2^College of Pharmacy, Hebei Medical University, Shijiazhuang, Hebei 050017, China; ^3^Department of Pharmacy, Hebei General Hospital, Hebei Key Laboratory of Clinical Pharmacy, Shijiazhuang, Hebei 050051, China; ^4^The second Hospital of Hebei Medical University, Shijiazhuang, Hebei 050000, China; ^5^Department of Pharmacology, Institution of Chinese Integrative Medicine, Key Laboratory of Neural and Vascular Biology, Ministry of Education, Key Laboratory of New Drug Pharmacology and Toxicology, Hebei Medical University, Shijiazhuang, Hebei 050017, China

**Keywords:** ADHD animal model, aniracetam, attention deficit hyperactivity disorder, behavior, TARP γ-8

## Abstract

Attention-deficit/hyperactivity disorder (ADHD) is a neurodevelopmental disorder that affects 8–12% of children globally. Hyperactivity-related behaviors, as well as inattention and impulsivity, are regarded as the nuclear symptoms of ADHD. At present, its etiologies and risk factors are unknown. Previous research linked TARP γ-8 deficiency to ADHD-like behaviors in mice, including hyperactivity, impulsivity, and memory deficits. Aniracetam, a nootropic drug, enhances cognition by modulating cholinergic activity and glutamate receptors, offering neuroprotective effects. This study examined TARP γ-8 knockout (KO) mice at 4 and 8 weeks, assessing behaviors through locomotor activity, cliff avoidance, novel object recognition, and contextual fear conditioning tests. TARP γ-8 KO mice exhibited hyperactivity, reduced recognition memory, and impaired short-term memory and long-term memory. Aniracetam administration improved these behavioral deficits, suggesting its potential as a therapeutic agent for ADHD. The findings align with ADHD’s pathophysiology, resembling the neurological impairments in TARP γ-8 KO mice. Aniracetam shows promise as a novel treatment for ADHD symptoms, highlighting its therapeutic value.

## Significance Statement

This study demonstrated for the first time that aniracetam works through glutamate receptors, including α-amino-3-hydroxy-5-methyl-4-isoxazolepropionic acid receptors (AMPARs), to successfully reduce the symptoms of attention deficit hyperactivity disorder (ADHD). According to the findings of our study, mice's behavior improved after receiving aniracetam. This achievement not only fills the gap in the treatment field of ADHD in terms of AMPARs but also provides a crucial basis for the optimization of subsequent treatment regimens. In addition, this study will encourage in-depth research into the pathophysiology of ADHD and medication development, giving medical advancements a boost.

## Introduction

Attention deficit hyperactivity disorder (ADHD) is a neurobehavioral disorder that is defined by persistent and maladaptive symptoms of hyperactivity/impulsivity and inattention. People with ADHD often have serious impairments in academic, social, and interpersonal functioning. ADHD is also associated with several comorbid conditions and disorders such as mood disorders, disruptive behavior disorders, and learning disabilities (Antshel et al., 2011). The prevalence of ADHD in children and adolescents is thought to be ∼3.4% worldwide (Polanczyk et al., 2015). Increasingly, ADHD is being recognized and diagnosed among young children between the ages of 2 and 6 years (preschoolers), and one of the most prevalent mental disorders in childhood and adolescence is anxiety. For children aged 4–6 years with ADHD, behavioral therapy or classroom interventions are the recommended initial treatment options. However, trends in the overall treatment for young children suggest that pharmacotherapy is more likely to be implemented than non-medication approaches (Visser et al., 2007). The current pharmacological treatment for ADHD is to increase catecholamine levels in the brain in order to improve focus, decrease distraction, and curb impulsive behavior (Briars and Todd, 2016). For the treatment of ADHD, methylphenidate (MPH) and atomoxetine (ATX) are the most often recommended drugs. They function by increasing extracellular norepinephrine (NE) and dopamine (DA; Storebo et al., 2018). However, there have been adverse effects associated with its use, including dyskinesia, anxiety, nausea, insomnia, hyperhidrosis, restlessness, disorientation, and dry eyes and mouth (de Sousa et al., 2022). Because they do not address the more extensive and, in particular, long-term treatment demands of many ADHD patients, their efficacy has been called into question (Posner et al., 2020).

Research on the pathophysiology and therapy of ADHD has sparked significant interest in the illness over the past few decades, much like other mental disorders. Studies in animal models have found that ADHD may be related to dopaminergic, noradrenergic, and serotonergic neurotransmission (Russell, 2011). However, the exact biological mechanisms of ADHD are not yet fully elucidated, and there remain no neurobiological diagnostic markers. It is believed that hypofunctional catecholamine systems in the striatum, nucleus accumbens, and prefrontal cortex (PFC) are involved in ADHD. However, recent clinical evidence has implicated glutamate, the main excitatory neurotransmitter in the CNS, in ADHD (Miller et al., 2014). In addition, mice lacking the function and expression of ionic glutamate receptors, such as α-amino-3-hydroxy-5-methyl-4-isoxazolepropionic acid receptors (AMPARs), subunits exhibit ADHD-like behaviors, such as hyperlocomotion (Aitta-Aho et al., 2019) and spatial working memory impairment (Reisel et al., 2002). Positive allosteric modulators of AMPARs inhibit locomotor hyperactivity in animal ADHD models and improve symptoms in adult subjects with ADHD (Adler et al., 2012). These results imply that AMPARs may be key contributors to ADHD phenotypes. TARP γ-8 is a critical protein for basal AMPAR expression, trafficking, localization during synaptic development, and plasticity. Recent studies have suggested that TARPγ-8 deficiency is closely associated with the development of ADHD. For instance, the behavioral results showed that adolescent TARPγ-8 KO mice exhibited MPH-sensitive ADHD-like phenotypes and the TARP γ-8 KO mice also displayed ADHD-like changes in the synaptosome proteomes in the PFC and hippocampus, which were rescued by MPH (Bai et al., 2022).

Aniracetam 1-[(4-methoxyphenyl)]-2-pyrrolidinone is a nootropic compound which has been the subject of extensive animal research and has been categorized as a piracetam analog and has minimal side effects (Wijayawardhane et al., 2008). Aniracetam has been demonstrated to improve long-term potentiation (LTP) in the hippocampus, raise excitatory postsynaptic potentials, and lengthen the decay period of excitatory postsynaptic currents (EPSCs; Satoh et al., 2022). On the other hand, it has been reported that aniracetam can enhance human cognitive function, including visual recognition, motor function, and general intellectual function (Elston et al., 2014). Another report has been studied for its anxiolytic effects in addition to its alleged cognitive enhancement. Aniracetam has proven effective in both human and nonhuman models of cognitive dysfunction (Reynolds et al., 2017). In both intact brain tissue and cultured neurons, aniracetam functions as a dual allosteric positive modulator of AMPARs and metabotropic glutamate receptors (Elston et al., 2014). In addition to directly enhancing glutamatergic synaptic transmission, aniracetam has been shown to activate nicotinic acetylcholine receptors in brain neurons and, via its cholinergic activity, indirectly boost dopaminergic neurotransmission (Elston et al., 2014). It is currently well accepted that aniracetam acts as a positive allosteric modulator on the AMPA subtype via a glutamatergic mode of action (Black, 2005, Vaglenova et al., 2008). Aniracetam slows both the deactivation and desensitization of AMPARs via stabilizing the glutamate-bound conformation, altering ion flux (Jin et al., 2005).

Thus, Wistar Kyoto rats (WKY) were selected as the normal control and TARP γ-8 KO mice were used as the ADHD model in this work. Young TARP γ-8 KO mice can mimic the ADHD-typical behaviors that closely resemble childhood symptoms of ADHD (Bai et al., 2022). In addition to the basal hyperactivity under a novel environment, we found that TARP γ-8 KO mice showed shortened jumping latency in cliff avoidance test, lower recognition index in object-based recognition test, and shorter freezing times (%) in the short-term memory (STM) and long-term memory (LTM) tests of the contextual fear conditioning (CFC) test. Thus, the current study sought to determine whether treating the TARP γ-8 KO mice with aniracetam will change their behavioral patterns, primarily their impulsivity, hyperactivity, anxiety-like behaviors, and processing/elimination of cognitive and memory impairment. In order to prevent and treat ADHD, such efforts can yield fresh concepts and new aims.

## Materials and Methods

### Mice and reagents

All animal studies were performed in accordance with the regulations of the ARRIVE guidelines for reporting experiments (“Animal research: reporting in vivo experiments: the ARRIVE guidelines,” 2010). The study was approved by the Ethics Committee of Hebei Medical University (Approval No.: IACUC-hebmu-2019001, Shijiazhuang, China). The TARP γ-8 KO mouse line was backcrossed to C57BL/6J mice a minimum of eight times before use in this study. Both homozygous TARP γ-8 KO mice and their wild-type (WT) littermates were obtained from crossbred heterozygous mice. Six mice of the same genotype were housed in one cage. All mice were maintained under constant temperature (22 ± 0.5°C) and humidity (50 ± 5%), using a 12 h light/dark cycle (lights on at 7:00 P.M.). Food and water were provided *ad libitum*. Four-to eight-week-old male mice (weight, 13.2–20.5 g) were used in all experiments. For the pharmacological rescue experiment, aniracetam (Sigma-Aldrich) was freshly prepared with 10% 2-hydroxypropyl-β-cyclodextrin (2-HP-β-CD) each time. TARP γ-8 KO and WT mice received an intraperitoneal (i.p.) injection of either 100 mg/kg aniracetam or vehicle (10% 2-hydroxypropyl-β-cyclodextrin) 30 min before the testing phase, respectively (Smith and Wehner, 2002, Bai et al., 2022).

### Behavioral tests

#### Open field test

An open field test is used to assess behavioral performance in ADHD model (Archer, 1973). This test was performed as previously described (Seibenhener and Wooten, 2015) with minor modifications. The apparatus consisted of four plastic chambers, each measuring 45 × 45 × 45 cm, with the center zone line located 10 cm from the edge. Each rat was gently placed in the center of the field and spontaneous locomotion was recording for 30 min. The surfaces of each box were cleaned and wiped with 75% ethanol at the end of every trial. Total exercise distance (cm), resting time (s), time spent in the central region (s), and time spent in edge area movement were recorded and analyzed by SMART Video Tracking software (Panlab; Li et al., 2017). Less time spent in the central zone reflects increased anxiety-like behaviors, and a greater total distance means more locomotion activity (Gong et al., 2022).

#### Cliff avoidance reaction

The cliff avoidance test was utilized to assess the impulsive behavior of mice (Li et al., 2017). The cliff avoidance test was evaluated with the use of a round platform (an inverted glass beaker with a diameter of 11 cm and a height of 15 cm; more than twice the body length of the animal) placed in the center of the open field apparatus (45 × 45 × 45 cm). The test was initiated by gently placing an animal on a platform such that the forelimbs approached its edge. If the animal fell from the platform, it was judged to have impaired cliff avoidance reaction (CAR). The amounts of leaping occurrences and the latency to the first jump were also recorded. Mice which fell from platforms were immediately and gently placed back on the platforms, and the test was continued until 10 min had elapsed. Mice which did not fall from platforms were tested for the same duration of time. After the test of each mouse, the surface of beaker was wiped by 75% ethanol. A mouse was judged to have an intact CAR if it did not jump off the platform during the 10 min test. The incidence of impaired CAR was calculated as a percentage index for each group (Yamashita et al., 2013):

CAR%=NumberofintactCARmice/Totalnumberoftestedmice×100%.

The saline or aniracetam was intraperitoneally administered 30 min before the onset of the cliff avoidance test.

#### Object recognition task

Based on the behavioral observation that rodents display greater interest and spend more time with newly encountered objects compared with familiar objects, the new object recognize test was developed to evaluate recognition memory and attention (Atalay et al., 2024). It was conducted in an open field made of white-painted wood measuring 45 × 45 × 45 cm. The general procedure consisted of three different sessions: habituation, training, and test. Each mouse was placed into the open-field box 5 min/day for 3 consecutive days (habituation phase). On the next day, two identical objects (A1 and A2) in the shape of a cube were placed in opposite and equidistant locations and each mouse were allowed to explore and familiarize the objects for 5 min (familiarization phase) and then returned to their home cages for a 1 h intertrial interval. After this time, the animals were returned for their home cage and the apparatus was cleaned. In the discrimination phase, one of the two identical objects (A1) was replaced with a novel object (B), and mice were allowed to explore freely for 5 min. Time spent exploring each object was recorded using SMART Video Tracking software (Partin, 2015). All familiar (A) and novel objects (B) were made of the same wooden material with a similar smell, color, and size but differences in shape. Exploration of an object was defined as directing the nose to the object at a distance of equal to or less than 2 cm or touching it with the nose. Novel object preference was determined as follows:

Discriminationratio(%)=(B−A2)/(B+A2)×100%.

A separate cohort of wild-type and TARP γ-8 KO mice were injected with saline or aniracetam 30 min before the familiarization phase for pharmacological rescue experiments.

#### Contextual fear conditioning

According to a previous study, a CFC procedure was provided (Gong et al., 2017). The general procedure consisted of three different sessions: habituation, training, and test. In the first session, habituation session, mice were briefly placed in the conditioning chamber and allowed to explore freely for 5 min daily for 3 d to acclimate to the testing environment. After 3 consecutive days of handling, the mice were placed in the training chamber on the day of the experiment and allowed to habituate for 120 s without any stimulation at training session. After this, the mice received a footshock (0.7 mA, 2 s) through the stainless-steel grid floor (Panlab) followed by a 58 s interval. The 2 s/58 s procedure was repeated three times, and the mice were returned to their home cages after the final 58 s period. All chambers were cleaned with 75% ethanol before and after all behavioral procedures. STM and LTM tests were conducted 1 and 24 h after training, when the subjects were returned to the training chambers. Freezing behavior, which is defined as the absence of any movement other than breathing, was evaluated over the course of five minutes in order to detect CFC (Kenney et al., 2012). Freezing time was analyzed using the PACKWIN animal behavior analysis system (Panlab), and memory score was determined as follows:

Freezingtime(%)=(Timespentfreezing)/(Totaltime)×100%.

For the pharmacological rescue experiment, saline or aniracetam was injected 30 min before the training phase.

#### Statistical analysis

All data are expressed as mean ± standard error values. Statistical analysis was performed using GraphPad Prism v8.0.2 (GraphPad). For comparisons of animal behavior, data normality was analyzed using the Shapiro–Wilk test. Normally distributed data were analyzed using unpaired Student's *t* test or two-way analysis of variance (ANOVA) followed by Bonferroni-corrected post hoc multiple comparisons. Non-normally distributed data were analyzed by nonparametric Mann–Whitney or Kruskal–Wallis tests followed by Dunn's multiple-comparisons tests. In the cliff avoidance test, the data for cumulative jumping events were compared by the log-rank (Mantel–Cox) test. *p* < 0.05 was set as the threshold for significance (*p < 0.05; **p < 0.01; ***p < 0.001).

## Results

### Aniracetam alleviated the hyperactivity and anxiety-like behaviors generated in TARP γ-8 KO adolescent mice

**Figure 1.**
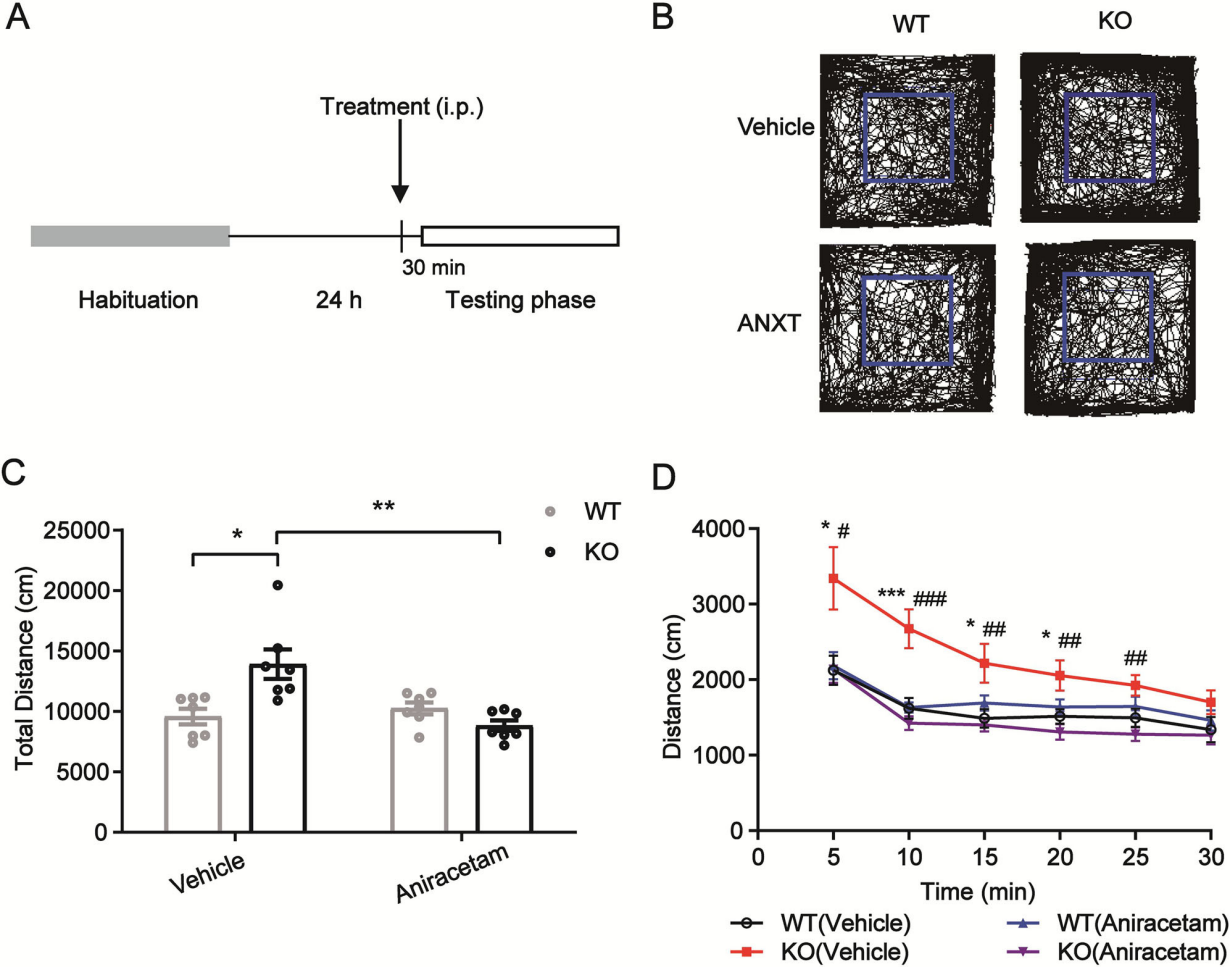
Adolescent TARP γ-8 KO mice treated with vehicle or aniracetam compared with WT mice showed different behavioral outcomes in the open field test. ***A–D***, Open field test in WT and KO mice treated with saline or aniracetam (100 mg/kg, i.p., 30 min before testing). ***A***, Schematic of experimental procedures. ***B***, Representative activity traces in open field. Inside of blue square represents central area, outside represents peripheral area. ***C***, Total distance traveled during 30 min: Kruskal–Wallis statistic (*H*_2_ = 14.63, *p* = 0.0022), Dunn's test(SAL + WT vs SAL + KO: *p* = 0.0282; SAL + KO vs ANXT + KO: *p* = 0.0016). ***D***, Distance traveled in the first 5 min: two-way ANOVA (*F*_strains(1,24)_ = 4.794, *p* = 0.0385; *F*_treatment(1,24)_ = 4.988, *p* = 0.0351; *F*_interaction(1,24)_ = 5.848, *p* = 0.0236), followed by Bonferroni's post hoc test(SAL + WT vs SAL + KO: *p* = 0.0186; SAL + KO vs ANXT + KO: *p* = 0.02). Distance traveled in the first 10 min: two-way ANOVA (*F*_strains(1,24) _= 15.54, *p* = 0.0006; *F*_treatment(1,24)_ = 7.301, *p* = 0.0124; *F*_interaction(1,24)_ = 16.05, *p* = 0.0005), followed by Bonferroni's post hoc test(SAL + WT vs SAL + KO: *p* = 0.0005; SAL + KO vs ANXT + KO: *p* < 0.0001). Distance traveled in 15 min: Kruskal–Wallis test (*H*_2_ = 12.47, *p* = 0.0059), Dunn's test(SAL + WT vs SAL + KO: *p* = 0.0381; SAL + KO vs ANXT + KO: *p* = 0.0062). Distance traveled in 20 min: two-way ANOVA (*F*_strains(1,24)_ = 5.501, *p* = 0.0276; *F*_treatment(1,24)_ = 0.6303, *p* = 0.4350; *F*_interaction(1,24)_ = 10.73, *p* = 0.0032), followed by Bonferroni's post hoc test(SAL + WT vs SAL + KO: *p* = 0.0497; SAL + KO vs ANXT + KO: *p* = 0.0034). Distance traveled in 25 min: two-way ANOVA (*F*_strains(1,24)_ = 4.139, *p* = 0.0531; *F*_treatment(1,24)_ = 0.0620, *p* = 0.8055; *F*_interaction(1,24)_ = 10.65, *p* = 0.0033), followed by Bonferroni's post hoc test(SAL + KO vs ANXT + KO: *p* = 0.006). All data are presented as mean ± SEM, *n* = 10 mice per group. *p < 0.05; **p < 0.01; ***p < 0.001.

Adult TARP γ-8 KO mice exhibit obvious hyperactivity in novel environments (Bai et al., 2022). We used the open field test on normal naive mice to see if aniracetam affected the hyperactivity and anxiety-like behaviors generated by TARP γ-8 KO mice. We evaluated locomotor activity in TARP γ-8 KO mice aged 4–8 weeks. As indicated by higher activity traces in the peripheral region (Fig. 1*B*), the teenage TARP γ-8 KO mice displayed minor anxiety symptoms in comparison with WT mice. A two-way ANOVA was used to examine the total distance in the open field test. Bonferroni's post hoc test showed that saline-treated TARP γ-8 KO mice showed higher locomotor activity than saline-treated WT mice, and these locomotor activity levels were considerably improved after aniracetam treatment (Fig. 1*C*).

As shown in Figure 1*D*, a two-way ANOVA analysis revealed a consistent pattern in the distance walked every 5 min over the 30 min test period (Fig. 1*D*). The results of Bonferroni's post hoc test showed that TARP γ-8 KO animals treated with saline had higher levels of spontaneous locomotor activity at 5, 10, and 20 min than WT mice treated with saline. These levels of spontaneous locomotor activities were considerably reduced with aniracetam administration. However, there was no significant difference during the distance traveled 25 min test between the saline-treated WT and TARP γ-8 KO groups, and Bonferroni's post hoc comparison showed significant distance traveled was rescued after aniracetam treatment (*p* < 0.01). The distance traveled test, which continued for 25 min, did not reveal any significant difference between the saline-treated WT and TARP γ-8 KO groups. However, Bonferroni's post hoc analysis revealed that the significant distance traveled was restored following aniracetam treatment (*p* < 0.01). The results demonstrated that the hyperactivity and anxiety-like behaviors that TARP γ-8 KO mice produced could be reduced in aniracetam-treated TARP γ-8 KO mice.

### The impulsivity that TARP γ-8 KO adolescent mice exhibited was reduced with aniracetam

**Figure 2.**
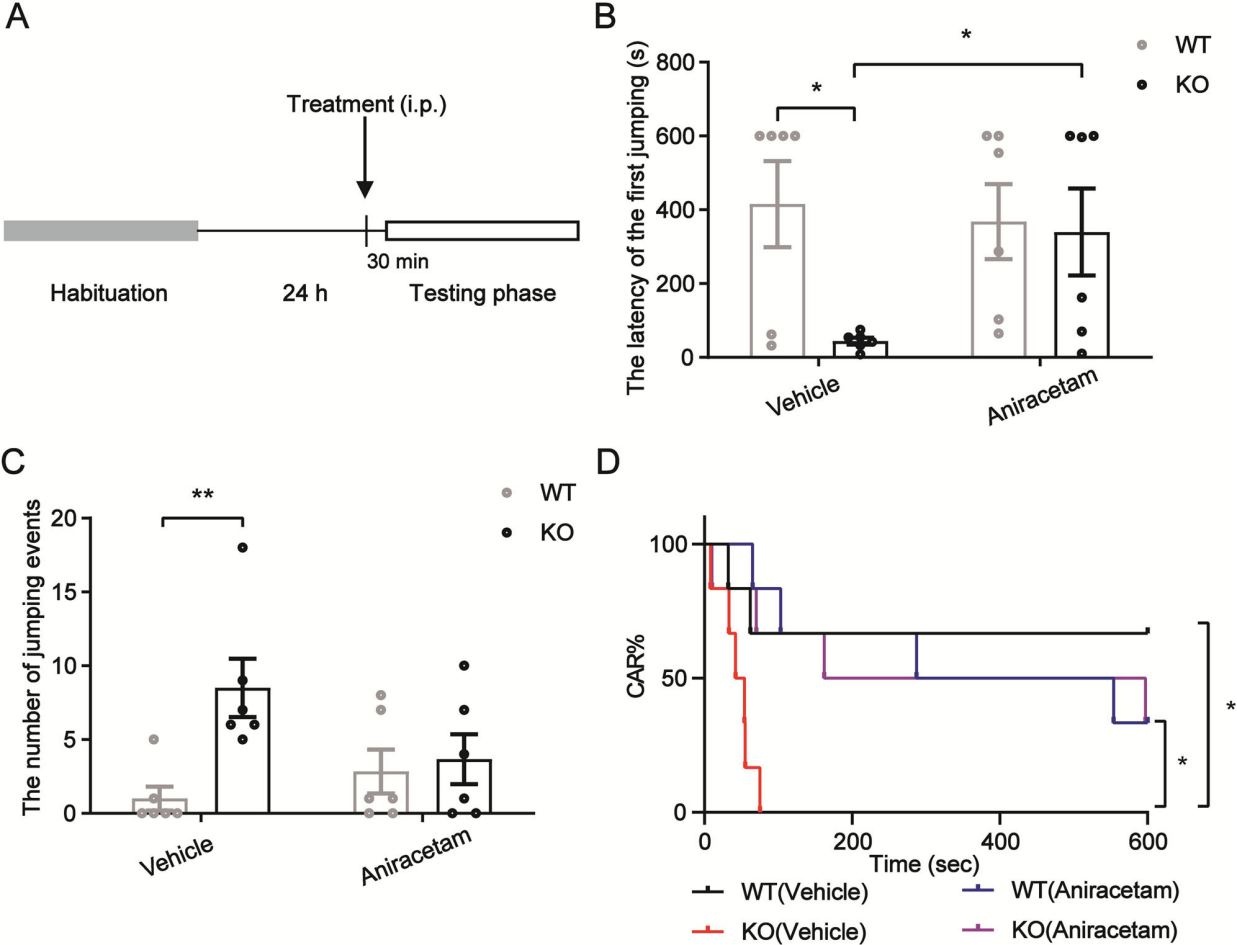
Adolescent TARP γ-8 KO mice treated with vehicle or aniracetam compared with WT mice showed different behavioral outcomes in the cliff avoidance reaction test (CAR). ***A***, Schematic of experimental procedures. ***B***, Time from initial placement on platform to first jump in 10 min: Kruskal–Wallis test (*H*_2_ = 7.992, *p* = 0.0462), Dunn's test (SAL + WT vs SAL + KO: *p* = 0.0141; SAL + KO vs ANXT + KO: *p* = 0.0459). ***C***, Number of jumping events in 10 min: Kruskal–Wallis test (*H*_2_ = 8.557, *p* = 0.0358),.Dunn's test (SAL + WT vs SAL + KO: *p* = 0.0037). ***D***, Percentage of mice with intact CAR: log-rank (Mantel–Cox) test: (SAL + WT vs SAL + KO: *p* = 0.0195; SAL + KO vs ANXT + KO: *p* = 0.0195). All data are presented as mean ± *SEM*, *n* = 10 mice per group*p < 0.05; **p < 0.01; ***p < 0.001.

CAR evaluates maladaptive impulsive rodent behavior. Impulsivity is a major characteristic of diseases such as ADHD (Yamashita et al., 2013). Next, we used the CAR test to look at how aniracetam affected the impulsive behavior of teenage TARP γ-8 KO mice. The latency of first jumping in the CAR test was analyzed using the Kruskal–Wallis test (*H*_2_ = 7.992, *p* = 0.0462). Dunn's post hoc test showed that saline-treated TARP γ-8 KO mice had a shorter latency of first jumping time than saline-treated WT mice and that this difference was significantly improved after aniracetam treatment (Fig. 2*B*). In addition, aniracetam did not affect the number of jumping events when compared with the saline-treated TARP γ-8 KO mice (Fig. 2*C*). However, the percentage of intact CAR mice (CAR%) varied considerably based on the log-rank test (Fig. 2*D*). Additionally, the Mantel–Cox comparison revealed that the CAR% (*p* < 0.05) of intact CAR mice in the TARP γ-8 KO group treated with saline was substantially lower than that of the aniracetam-treated group (*p* = 0.0195).

### The defects in recognition memory caused by TARP γ-8 KO adolescent mice are improved by aniracetam

**Figure 3.**
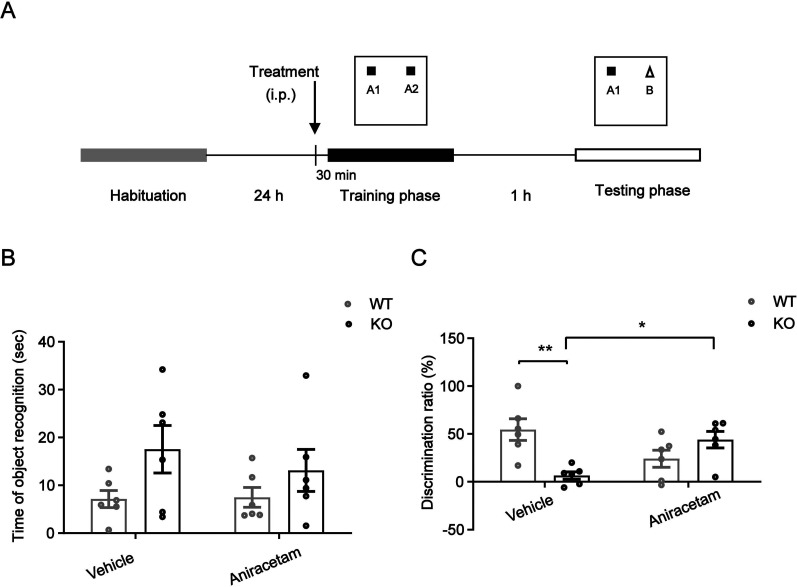
Adolescent TARP γ-8 KO mice treated with vehicle or aniracetam compared with WT mice showed different behavioral outcomes in the novel object recognition test. ***A***, Schematic of experimental procedures. ***B***, Total time exploring novel and familiar objects in the testing session. ***C***, Discrimination ratio in testing session: two-way ANOVA (*F*_strains(1,24)_ = 0.1671, *p* = 0.6871; *F*_treatment(1,24)_ = 2.653, *p* = 0.1190; *F*_interaction(1,24)_ = 15.53, *p* = 0.0008), Bonferroni's post hoc test (SAL + WT vs SAL + KO: *p* = 0.0049; SAL + KO vs ANXT + KO: *p* = 0.0358). All data are presented as mean ± SEM, *n* = 10 mice per group; *p < 0.05; **p < 0.01; ***p < 0.001.

Individuals with ADHD exhibit cognitive deficits such as impaired memory (Won et al., 2011). The object recognition task was proposed to assess discrimination and memory performance, in which measurements are based on the difference of time spent on familiar versus novel objects (Bouchatta et al., 2018). Thus, we assessed also cognitive functions through memory and learning in adolescent TARP γ-8 KO mice. In the object recognition test, the total time for object recognition did not differ significantly between the saline-treated WT and TARP γ-8 KO groups (Fig. 3*B*). Additionally, aniracetam did not affect the total time for object recognition when compared with the saline-treated TARP γ-8 KO mice. However, two-way ANOVA showed a significant difference in the discrimination ratio (%) (Fig. 3*C*), and Bonferroni's post hoc comparison demonstrated a significant impairment in recognition function (*p* < 0.01) in the TARP γ-8 KO group treated with saline, which was clearly saved after aniracetam treatment (*p* = 0.0358). The impulsivity displayed by adolescent mice lacking TARP γ-8 was decreased when treated with aniracetam.

### Aniracetam enhanced the recall of contextual terror produced by TARP γ-8 KO adolescent mice

**Figure 4.**
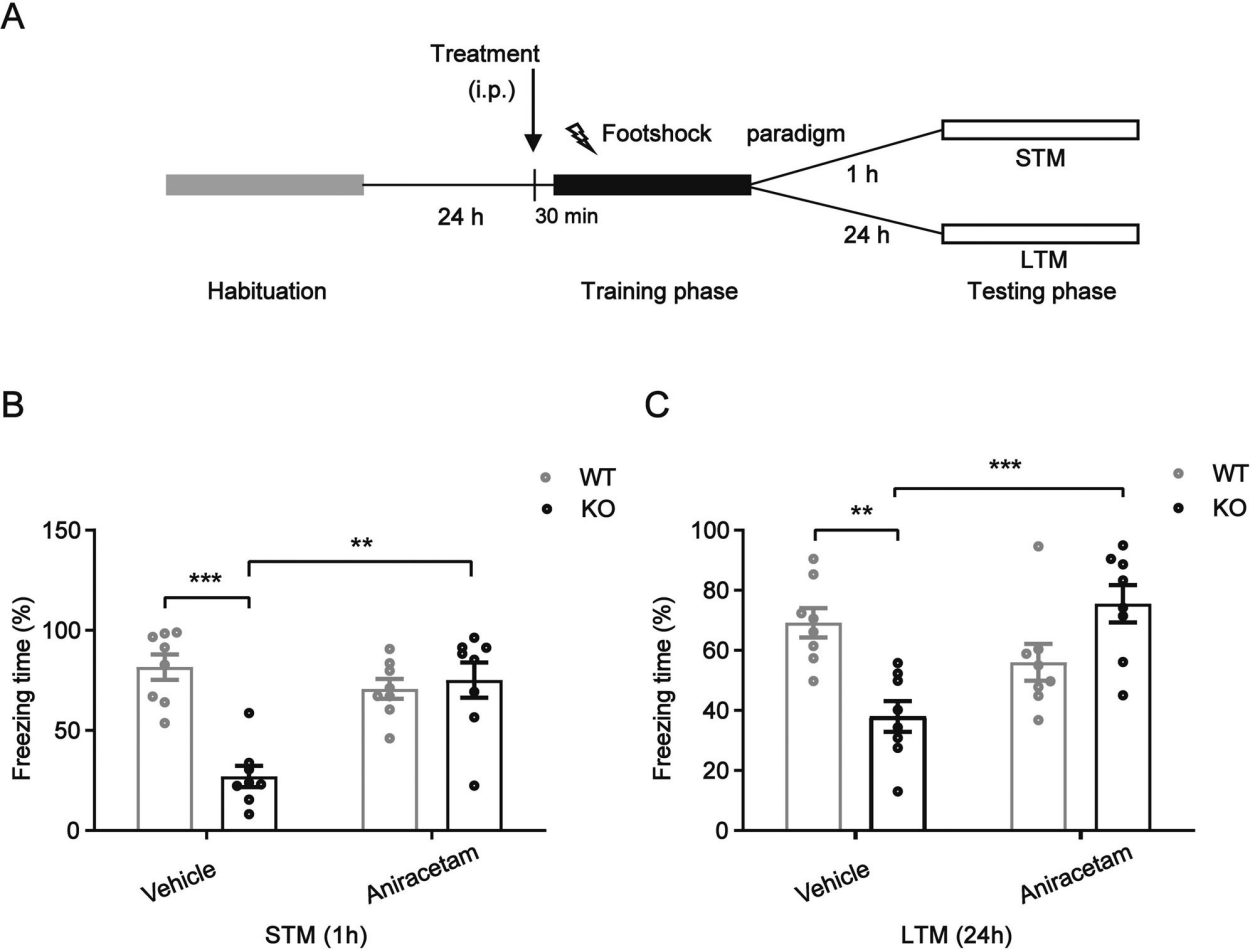
Adolescent TARP γ-8 KO mice treated with vehicle or aniracetam compared with WT mice showed different behavioral outcomes in the CFC test. Short-term memory (STM) and long-term memory (LTM) were assessed by measuring freezing behavior 1 and 24 h after training, respectively. ***A***, Schematic of experimental procedures. ***B***, Freezing time (%) of STM: Kruskal–Wallis test (*H*_2_ = 15.80, *p* = 0.0012), Dunn's test (SAL + WT vs SAL + KO: *p* = 0.0003; SAL + KO vs ANXT + KO: 0.0017); Freezing time (%) of LTM: two-way ANOVA (*F*_strains(1,24)_ = 4.714, *p* = 0.0385; *F*_treatment(1,24)_ = 1.080, *p* = 0.3075; *F*_interaction(1,24)_ = 20.40, *p* = 0.0001), Bonferroni's post hoc test(SAL + WT vs SAL + KO: 0.003; SAL + KO vs ANXT + KO: 0.0003). All data are presented as mean ± SEM, *n* = 10 mice per group; *p < 0.05; **p < 0.01; ***p < 0.001.

Previous studies have reported spontaneously hypertensive rats (SHRs) exhibit a deﬁcit in CFC (Calzavara et al., 2011). Fear conditioning is a widely used paradigm in the study of the neurological underpinnings of emotion (Calzavara et al., 2009). Therefore, we assessed the memory for contextual fear in teenage TARP γ-8 KO mice. The duration of freezing, which is characterized as the animal's total immobility without any vibrations or smelling, was measured for 5 min. Tests for STM and LTM were conducted 1 and 24 h following the training sessions, respectively. In the CFC test, the Kruskal–Wallis statistic (*H*_2_ = 15.80, *p* = 0.0012) was used to analyze the STM freezing time (%). Dunn's post hoc test showed that saline-treated TARP γ-8 KO mice had shorter freezing times than saline-treated WT mice and that these differences were significantly improved after aniracetam treatment (Fig. 4*B*). A similar tendency was additionally observed in LTM (Fig. 4*C*), which was examined using two-way ANOVA analysis. Bonferroni's post hoc test demonstrated that LTM was considerably worse in saline-treated TARP γ-8 KO mice as opposed to saline-treated WT mice, and post hoc analysis indicated that aniracetam greatly recovered LTM.

## Discussion

The most prevalent neurodevelopmental illness in children is ADHD. It is also one of the most common chronic illnesses affecting school-age children. Nevertheless, the pharmacological mechanism and etiology of ADHD remain unclear. The brain's inadequate catecholamine system may be the cause of hyperactivity and inattention, consistent with accumulated research. Monoamine neurotransmitters, such as DA and NE, play an important role in keeping brain excitability and alertness, which are considered to be the biochemical mechanism of attention (Chen et al., 2017). Monoamine neurotransmitters have been implicated in the pathophysiology of ADHD in numerous clinical trials (Cortese, 2012, Karmakar et al., 2014, 2016). Nowadays, almost every medication used to treat ADHD has something to do with altering monoamine neurotransmitters. Furthermore, an abnormal balance of neural excitation and inhibition (E/I) arises due to the contribution of GABA and glutamate, resulting in a dysfunction of information transmission between the excitatory and inhibitory synapses (Mamiya et al., 2021, Xi and Wu, 2021). The diagnostic of ADHD, like other psychiatric disorders, relies on behavioral assessment. The clinical symptoms of ADHD, especially the three main ones of hyperactivity, impulsivity, and attention impairment, must be replicated in animal models. The study of human psychiatric problems can benefit from the use of certain criteria from animal models, including causation, genetic similarities, physiological mechanisms, and treatment. Three forms of validity were chosen: predictive, face, and construct (Kim et al., 2024). SHRs are one of the most studied animal models of ADHD (Wang et al., 2017, Leffa et al., 2018). SHR possess face validity in that they are hyperactive, impulsive, and inattentive compared with WKY controls, suggesting that this animal could be used as a model for ADHD (Kim et al., 2024). Regarding predictive validity, methylphenidate reduces the abovementioned ADHD-like behaviors in SHR (Regan et al., 2022). Furthermore, it has been demonstrated that psychostimulants and the alpha2-adrenoreceptor agonist guanfacine can occasionally reduce hyperactivity, impulsivity, and inattention in SHR, which lends some support to the model's predictive validity (Myers et al., 1982, Sagvolden, 2006). SHR neuropathological changes are similar to those observed in ADHD patients. Previous references demonstrated that the prefrontal caudate-putamen cortex and NAc, two regions that are obviously impacted in ADHD, were the sites where the reduced release of DA was found in SHR (Leffa et al., 2018, 2019). Overall, the SHR model has been thoroughly studied and exhibits a number of traits associated with ADHD, including impulsivity, working memory impairments, hyperactivity, and in certain tests, decreased attention. The SHR is the most used model of ADHD due to its predictive validity, face validity, and partial construct validity. Previous research has shown that TARP γ-8 KO in teenage mice is utilized as a model for ADHD because the mice exhibit hyperactive behavior even in familiar settings (Bai et al., 2022). Additionally, the important ADHD medicine MPH, which is frequently used in other ADHD animal models, was able to repair the main ADHD-like symptoms and changed synaptosomal proteins in TARP γ-8 KO mice (Won et al., 2011, Li et al., 2017). TARP γ-8 KO mice's hippocampi showed malfunction of the AMPA glutamate receptor complex according to synaptosome proteomic profiling. In the prefrontal cortices of TARP γ-8 KO mice, proteomic analysis also demonstrated disruption of glutamatergic and dopaminergic transmissions (Bai et al., 2022). Based on the above, TARP γ-8 KO meets the requirements (facial, constructal, and predictive validity) for ADHD animal models, suggesting that teenage TARP γ-8 KO mice could be used as a murine model of ADHD (de la Pena et al., 2018).

Psychostimulants including MPH and amphetamine derivatives have also been recommended as the first-line treatment according to clinical guidelines for school-aged children diagnosed with ADHD (Chiu et al., 2023). MPH can be viewed as a dopamine reuptake inhibitor, which facilitates dopaminergic neurotransmission at the dopamine transporter, and elicits little presynaptic dopamine release. In contrast, amphetamines are believed to promote neurotransmitter release via reverse transport and prevent dopamine and norepinephrine from being reabsorbed into the presynaptic cell (Faraone and Buitelaar, 2010). By enhancing the impact of dopamine and norepinephrine, psychostimulants increase the efficiency of PFC activity and optimize executive and attentional function in patients suffering from ADHD (Mechler et al., 2022). Nevertheless, there are a few possible adverse effects of MPH and amphetamine, including reduced appetite, increased aggression, and physical symptoms like headaches and gastrointestinal issues (Nanda et al., 2023). Furthermore, current research supports the idea that prolonged exposure to stimulant drugs may raise the risk of psychosis because of their dopaminergic action (Krinzinger et al., 2019). Future study of different medications is promising in the realm of ADHD as this is a heterogenous illness and therefore heterogenous treatments should be available. Consequently, the ideal medicine would be one that targets numerous receptors at once, has no potential for addiction, and has no cardiac side effects while providing the same level of symptom relief as stimulants (O'Connor et al., 2023). Aniracetam is a cognitive enhancer belonging to the pyrrolidinone class, known for its therapeutic use in managing the behavioral and psychological symptoms of poststroke dementia and Alzheimer's disease. It has not been used for ADHD research so far. By strengthening the bond between glutamate and the ligand-binding domain, aniracetam, a positive allosteric modulator, stabilizes the glutamate-bound state and decreases the rate of AMPAR deactivation. Research has indicated that aniracetam lengthened the decay period of mEPSCs and raised the single channel mean open times. It is reasonable to believe that increased synaptic activity in the presence of aniracetam may trigger intracellular signaling cascades, which would alter AMPAR subunit expression and posttranslationally modify them (Wijayawardhane et al., 2008). The advantageous effects of aniracetam on neuronal function may be mediated through a variety of ways. Aniracetam has been demonstrated to improve LTP in addition to its well-established impact on AMPA receptor desensitization (Baranova et al., 2006). Furthermore, this nootropic chemical pyrrolidinone improves cognitive function via modifying glutamatergic transmission in the hippocampal region (Wijayawardhane et al., 2007). Aniracetam has been shown in prior clinical trials to be safe and effective in treating emotional dysregulation, sleep issues, behavioral issues, and cognitive impairment in people with Alzheimer's or cerebrovascular illness. Previous studies have demonstrated that aniracetam has a rehabilitative effect in animal models of memory impairment. Aniracetam helps older adults’ memory function (Bartolini et al., 1996), helps restore lost memory function after brain trauma (Baranova et al., 2006), and reverses impairment produced by scopolamine during a passive avoidance task (Martin et al., 1995). Likewise, when given before scopolamine or CO2 disruption, aniracetam has a protective effect (Phillips et al., 2019). Recent preclinical studies have demonstrated aniracetam's unique therapeutic properties linked to functional recovery in a variety of animal models of cerebral dysfunctional diseases. These consist of impulsive behavior, hyperactivity, fear and anxiety, hypo-vigilance arousal, hypo-attention (Nakamura, 2002). There is substantial evidence that aniracetam improves cognitive function in affected (i.e., brain damaged) subjects but seems to have no effect on healthy subjects (Elston et al., 2014). According to a few recent research, aniracetam had no therapeutic advantage in healthy mice across a range of learning and memory paradigms (Elston et al., 2014, Reynolds et al., 2017). Research demonstrates unequivocally that oral administration of 50 mg/kg of aniracetam repeatedly does not alter learning and memory, anxiety, movement, or repetitive behavior (Elston et al., 2014). According to previous studies, aniracetam has been shown to be effective in treating the three primary symptoms of ADHD—inattention, hyperactivity, and impulsivity—as well as the concomitant symptoms of anxiety, depression, dysthymia, and social failure (issues with sleep and impairments). As a result, we evaluated in the present study how the cognition-improving drug aniracetam affected the behavioral functioning of those with ADHD. The research and development of therapeutic medications for ADHD now have fresh perspectives and theoretical underpinnings thanks to this study.

In this work, we evaluated the behavioral alterations in adolescent TARP γ-8 KO mice before and after pharmaceutical intervention using behavioral tests. Aniracetam administration dramatically decreased the levels of spontaneous locomotor activity in the open field test, as well as the total distance traveled and the distance traveled every 3 min throughout the 30 min test in teenage TARP γ-8 KO mice, as illustrated in Figure 1. These suggest that aniracetam can help alleviate the hyperactivity and anxiety-like behaviors that these mice exhibit when compared with WT mice. Furthermore, in the cliff avoidance test, teenage TARP γ-8 KO mice exhibited much shorter leaping latency in comparison with more frequent jumping episodes with WT mice. The impulsivity of the teenage TARP γ-8 KO mice was significantly improved in Figure 2 as a result of aniracetam's ability to shorten their latency, minimize their leaping episodes, and increase their CAR%. Similarly, we tried to use the novel object recognition test to see if aniracetam may lessen the attention and recognition memory deficits in TARP γ-8 KO mice. In this current investigation, we found that aniracetam mitigated the recognition memory deficits in TARP γ-8 KO mice (Fig. 3*C*). Consequently, our findings suggest that aniracetam may enhance the establishment of novel object recognition memory. Finally, we evaluated the CFC test, a form of classical conditioning that allows for the analysis of fear reactions to a context and a cue. We discovered that in teenage TARP γ-8 KO mice, aniracetam repaired the defects in contextual fear memories. As established, animal models such as the TARP γ-8 KO mouse provide an avenue for evaluating ADHD progression and treatment. The effects of aniracetam in teenage TARP γ-8 KO mice that focus on neurological traits are not yet covered in any study. Aniracetam's impacts on the mouse model's behavioral phenotype have been assessed in this study through preliminary research. The results, in spite of several drawbacks, show that the aniracetam used in this study are highly effective in changing the behavior of TARP γ-8 KO mouse, and further investigation is necessary. Therefore, the present study suggests that aniracetam could be a novel agent for the treatment of ADHD.

## Data Availability

The datasets used and analyzed during the current study are available from the corresponding author upon reasonable request.

## Synthesis

Reviewing Editor: Christine Portfors, Washington State University Decisions are customarily a result of the Reviewing Editor and the peer reviewers coming together and discussing their recommendations until a consensus is reached. When revisions are invited, a fact-based synthesis statement explaining their decision and outlining what is needed to prepare a revision will be listed below. The following reviewer(s) agreed to reveal their identity: Rupesh Chillale.

The reviewers find this manuscript to provide an important addition to the literature. Methodological and conceptual limitations in the manuscript, however, reduce the clarity and impact of the study.

Major Comments:

1. While TARP γ-8 KO mice mimic certain ADHD-like behaviors, the generalizability to human ADHD remains limited. Many symptoms of ADHD are complex and multifactorial, and this single-gene knockout model may not represent this heterogeneity. This should be addressed in the discussion section.

2. Previous studies often use spontaneously hypertensive rats (SHRs), which might provide more direct parallels to hyperactivity and impulsivity. How the results of the TARP γ-8 KO mice show on these test?

3. The authors could compare their findings with these established models to contextualize the relevance of TARP γ-8 KO mice.

4. The study does not explicitly report power calculations or rationale for the chosen sample size. This raises questions about the robustness of the findings, particularly for complex behavioral data with high variability. The authors should comment on this aspect.

5. ADHD is characterized by deficits in executive function, attention, and impulsivity. While impulsivity and anxiety are well-assessed, the study does not measure attention directly, a critical aspect of ADHD.Broader behavioral paradigms, such as the five-choice serial reaction time task (5-CSRTT), could assess attention more robustly. The authors can compare their results with these behavioral tests

6. Prior studies have demonstrated Aniracetam's cognitive-enhancing effects in Alzheimer's and stroke models but with inconsistent results in normal animals. For example, Elston et al. (2014) found no significant cognitive improvement in healthy mice and the authors could strengthen their conclusions by contrasting their results with studies like Wijayawardhane et al. (2008), which highlight Aniracetam's impact on synaptic plasticity and AMPA receptor modulation.

7. The study aligns with research suggesting glutamate dysregulation in ADHD

Minor corrections:

1. Line 58, "lacking the function" not "lacking he function"

2. Lot of places there is no gap between words and citation brackets, please check citations in paragraph.

## Author Response

Dear Editor, We are very grateful for the time and effort of the editor and reviewers in evaluating our manuscript "Aniracetam ameliorates attention deficit hyperactivity disorder behavior in adolescent mice" (Manuscript ID: eN-CFN-0578-24). Those comments are all valuable and very helpful for revising and improving our paper, as well as the important guiding significant to our researches. In response to the reviewers' comments and suggestions, we have thoroughly revised our paper. Below we included a detailed point-by-point response to the reviewers' comments.

Response to reviewer 1:

1) While TARP γ-8 KO mice mimic certain ADHD-like behaviors, the generalizability to human ADHD remains limited. Many symptoms of ADHD are complex and multifactorial, and this single-gene knockout model may not represent this heterogeneity. This should be addressed in the discussion section.

Response: Thank you for pointing out this problem. The animal models of psychiatric disorders are usually evaluated and validated concerning three criteria: (1) face validity, (2) construct validity, and (3) predictive validity (Kim et al.,2024). Previous research has shown that TARP γ-8 KO in teenage mice is utilized as a model for ADHD because the mice exhibit hyperactive behavior even in familiar settings (Bai et al.,2022). Additionally, the important ADHD medicine MPH, which is frequently used in other ADHD animal models, was able to repair the main ADHD-like symptoms and changed synaptosomal proteins in TARP γ-8 KO mice (Li et al.,2017, Won et al.,2011). TARP γ-8 KO mice's hippocampi showed malfunction of the AMPA glutamate receptor complex according to synaptosome proteomic profiling. In the prefrontal cortices of TARP γ-8 KO mice, proteomic analysis also demonstrated disruption of glutamatergic and dopaminergic transmissions (Baranova et al.,2006). Given that they meet the requirements (facial, constructal, and predictive validity) for ADHD animal models, suggesting that teenage TARP γ-8 KO mice could be used as a murine model of ADHD (de la Pena et al.,2018). We added the following sentence "The study of human psychiatric problems can benefit from the use of certain criteria from animal models, including causation, genetic similarities, physiological mechanisms, and treatment. Three forms of validity were chosen: predictive, face, and construct (Kim et al.,2024)" and "Given that they meet the requirements (facial, constructal, and predictive validity) for ADHD animal models" in the first paragraph of the discussion section, sentences on page 8, lines 279-282 and304-305.

2) Previous studies often use spontaneously hypertensive rats (SHRs), which might provide more direct parallels to hyperactivity and impulsivity. How the results of the TARP γ-8 KO mice show on these tests? Response: Thanks for your advice. In this test, aniracetam administration dramatically decreased the levels of spontaneous locomotor activity in the open field test, as well as the total distance traveled and the distance traveled every five minutes throughout the 30-minute test in teenage TARP γ-8 KO mice. These suggests that aniracetam can help alleviate the hyperactivity and anxiety-like behaviors that these mice exhibit when compared to WT mice. Furthermore, in the cliff avoidance test, teenage TARP γ-8 KO mice exhibited much shorter leaping latency as comparison to more frequent jumping episodes with WT mice. The impulsivity of the teenage TARP γ-8 KO mice was significantly improved as a result of aniracetam's ability to shorten their latency, minimize their leaping episodes, and increase their CAR%. Thus, we found that aniracetam improved the hyperactivity and impulsivity in TARP γ-8 KO mice.

3) The authors could compare their findings with these established models to contextualize the relevance of TARP γ-8 KO mice.

Response: Thank you very much for your professional and constructive comment.

Spontaneously hypertensive (SH) rats are one of the most studied animal models of ADHD (Wijayawardhane et al.,2008). Thus, we made the decision to compare TARP γ-8 with SHR animal models. We added the following sentence "Spontaneously hypertensive (SH) rats are one of the most studied animal models of ADHD (Leffa et al.,2018, Wang et al.,2017). SHR possess face validity in that they are hyperactive, impulsive, and inattentive compared with WKY controls, suggesting that this animal could be used as a model for ADHD (Kim et al.,2024). Regarding predictive validity, methylphenidate reduces the above-mentioned ADHD-like behaviors in SHR (Regan et al.,2022). Furthermore, it has been demonstrated that psychostimulants and the alpha2-adrenoreceptor agonist guanfacine can occasionally reduce hyperactivity, impulsivity, and inattention in SHR, which lends some support to the model's predictive validity (Myers et al.,1982, Sagvolden,2006). SHR neuropathological changes are similar to those observed in ADHD patients .Previous references demonstrated that the prefrontal caudate-putamen cortex and NAc, two regions that are obviously impacted in ADHD, were the sites where the reduced release of DA was found in SHR (Leffa et al.,2019, Leffa et al.,2018). Overall, the SHR model has been thoroughly studied and exhibits a number of traits associated with ADHD, including impulsivity, working memory impairments, hyperactivity, and in certain tests, decreased attention. The SHR is the most used model of ADHD due to its predictive validity, face validity, and partial construct validity" in the first paragraphs of the discussion section, sentences on page 8, lines282-296.

4) The study does not explicitly report power calculations or rationale for the chosen sample size. This raises questions about the robustness of the findings, particularly for complex behavioral data with high variability. The authors should comment on this aspect.

Response: We sincerely appreciate your constructive suggestion about increasing the sample size. We fully understand the significance of a larger sample size in enhancing the reliability and generalizability of research findings. However, after careful evaluation, we are unable to increase the sample size for the following reasons. First, the research subjects in our study, TARP γ-8 KO mouse, are extremely scarce. Second, our research explores a relatively new area in ADHD, and to the best of our knowledge, there is a scarcity of directly relevant published works. However, to strengthen the theoretical basis and the credibility of our experiment, we have cited (Weiss et al.,2007), which makes use of KO+ Sacc (n = 9) and KO+ Nic (n = 8), as well as WT+ Sacc (n = 10) and WT+ Nic (n = 10). We make reference to (Song et al.,2020), which is broken down into four groups: n = 4-6 for each group. Each of the four groups (n = 10) in (Yuan et al.,2019) is referred to. We believe that these efforts can compensate for the relatively small sample size to some extent. Thank you again for your valuable feedback, and we are looking forward to your further comments.

5) ADHD is characterized by deficits in executive function, attention, and impulsivity. While impulsivity and anxiety are well-assessed, the study does not measure attention directly, a critical aspect of ADHD. Broader behavioral paradigms, such as the five-choice serial reaction time task (5-CSRTT), could assess attention more robustly. The authors can compare their results with these behavioral tests Response: We appreciate your comments very much. We agreed with your suggestions very much. Since attention deficit is one of the core symptoms of ADHD, attention deficit behavior should be assessed in standard ADHD animal models. Several behavioral test methods have been developed for experimental animal studies of attention in ADHD models, including 5-choice serial reaction time task (5-CSRTT) (Custodio et al.,2018, Li et al.,2017) and novel object recognition (NOR) test (Custodio et al.,2018, de la Pena et al.,2018, Toriumi et al.,2018). The 5-CSRTT is often used to assess sustained attention, which refers to the extended selective engagement of a behavioral or neural system that primarily enhances information processing in that system (Bartolini et al.,1996). And the NOR task could be used to assess selective attention, which involves the selection of a specific object or spatial location as the focus of attention (Levin et al.,2011, Reynolds,2015). In these behavioral works, attention and recognition memory are tightly, inseparably, coupled to work together. In standard ADHD models, a fully comprehensive study of attentional function should include 5-CSRTT and NOR evaluations, but most research do not fully (Drerup et al.,2010, Toriumi et al.,2018, Won et al.,2011, Zimmermann et al.,2015). Unfortunately, we only carried out the NOR task and did not continue to do the 5-CSRTT in γ-8 KO mice studies due to the lack of corresponding experimental equipment in our laboratory. However, we provided solid evidence for cognitive impairment, and hyperactivity, impulsivity, anxiety, memory impairment in γ-8 KO mice, which met the face validity in the animal model of ADHD, especially for ADHD of predominantly hyperactive/impulsive presentation.

6）Prior studies have demonstrated Aniracetam's cognitive-enhancing effects in Alzheimer's and stroke models but with inconsistent results in normal animals. For example, Elston et al. (2014) found no significant cognitive improvement in healthy mice and the authors could strengthen their conclusions by contrasting their results with studies like Wijayawardhane et al. (2008), which highlight Aniracetam's impact on synaptic plasticity and AMPA receptor modulation.

Response: Thank you very much for your constructive comment. There is growing evidence supporting the restorative effect of aniracetam in animal models of disease or injury. Aniracetam helps older adults' memory function (Bartolini et al.,1996), helps restore lost memory function after brain trauma (Baranova et al.,2006), and reverses impairment produced by scopolamine during a passive avoidance task (Martin et al.,1995). There is substantial evidence that aniracetam improves cognitive function in affected (i.e., brain damaged) subjects but seems to have no effect on healthy subjects (Elston et al.,2014). A few recent studies have found that aniracetam conveys no therapeutic benefit in healthy mice with respect to a variety of learning and memory paradigms (Elston et al.,2014, Reynolds et al.,2017). There is increasing evidence that aniracetam has a positive interaction with central glutamatergic systems, potentiating endogenous glutamate release (Togashi et al.,2002). Apart from inhibiting AMPA receptor desensitization, aniracetam also promotes the release of several other neurotransmitters that may improve neuronal function and aid in cognitive recovery. Aniracetam's beneficial effects on neuronal function could be mediated via multiple mechanisms of action. In addition to aniracetam's well-documented effect on AMPA receptor desensitization, aniracetam has been shown to enhance long-term potentiation (LTP) (Baranova et al.,2006). We added the following sentence" Research has indicated that aniracetam lengthened the decay period of mEPSCs and raised the single channel mean open times. It is reasonable to believe that increased synaptic activity in the presence of aniracetam may trigger intracellular signaling cascades, which would alter AMPAR subunit expression and post translationally modify them (Wijayawardhane et al.,2008). The advantageous effects of aniracetam on neuronal function may be mediated through a variety of ways. Aniracetam has been demonstrated to improve long-term potentiation (LTP) in addition to its well-established impact on AMPA receptor desensitization (Baranova et al.,2006). Previous studies have demonstrated that aniracetam has a rehabilitative effect in animal models of memory impairment. Aniracetam helps older adults' memory function (Bartolini et al.,1996), helps restore lost memory function after brain trauma (Baranova et al.,2006), and reverses impairment produced by scopolamine during a passive avoidance task (Martin et al.,1995). Likewise, when given before scopolamine or CO2 disruption, aniracetam has a protective effect (Phillips et al.,2019). There is substantial evidence that aniracetam improves cognitive function in affected (i.e., brain damaged) subjects but seems to have no effect on healthy subjects(Elston et al.,2014). According to a few recent research, aniracetam had no therapeutic advantage in healthy mice across a range of learning and memory paradigms (Elston et al.,2014, Reynolds et al.,2017). Research demonstrates unequivocally that oral administration of 50 mg/kg of aniracetam repeatedly does not alter learning and memory, anxiety, movement, or repetitive behavior (Elston et al.,2014) " in the second paragraph of the discussion section, the sentence on page 9, lines 329-353.

7) The study aligns with research suggesting glutamate dysregulation in ADHD Response: Thank you very much for your advice. Recent research has linked ADHD to glutamate, the primary excitatory neurotransmitter in the central nervous system (Miller et al.,2014). In addition, mice lacking the function and expression of ionic glutamate receptors, such as α-amino-3-hydroxy-5-methyl-4-isoxazolepropionic acid receptors (AMPARs), subunits exhibit ADHD-like behaviors, such as hyperlocomotion (Aitta-Aho et al.,2019) and spatial working memory impairment (Reisel et al.,2002). TARP γ-8 is a critical protein for basal AMPAR expression, trafficking, localization during synaptic development, and plasticity. Recent studies have suggested that TARPγ-8 deficiency is closely associated with the development of ADHD (Bai et al.,2022).

Minor Comments:

1. Line 58, "lacking the function" not "lacking he function" 2. Lot of places there is no gap between words and citation brackets, please check citations in paragraph.

Thanks for your careful checks. We are sorry for our carelessness. Based on your comments, we have made the corrections to make the word harmonized within the whole manuscript.

We tried our best to improve the manuscript and made some changes marked in yellow in revised paper which will not influence the content and framework of the paper. We appreciate for Reviewers' warm work earnestly, and hope the correction will meet with approval. Once again, thank you very much for your comments and suggestions.

References:

Aitta-Aho, T.; Maksimovic, M.; Dahl, K.; Sprengel, R.; Korpi, E. R.(2019).Attenuation of Novelty-Induced Hyperactivity of Gria1-/- Mice by Cannabidiol and Hippocampal Inhibitory Chemogenetics.FRONT PHARMACOL 10:309.

Bai, W. J.; Luo, X. G.; Jin, B. H.; Zhu, K. S.; Guo, W. Y.; Zhu, X. Q.; Qin, X.; Yang, Z. X.; Zhao, J. J.; Chen, S. R.; Wang, R.; Hao, J.; Wang, F.; Shi, Y. S.; Kong, D. Z.; Zhang, W.(2022).Deficiency of transmembrane AMPA receptor regulatory protein gamma-8 leads to attention-deficit hyperactivity disorder-like behavior in mice.ZOOL RES 43:851.

Baranova, A. I.; Whiting, M. D.; Hamm, R. J.(2006).Delayed, post-injury treatment with aniracetam improves cognitive performance after traumatic brain injury in rats.J NEUROTRAUM 23:1233.

Bartolini, L.; Casamenti, F.; Pepeu, G.(1996).Aniracetam restores object recognition impaired by age, scopolamine, and nucleus basalis lesions.PHARMACOL BIOCHEM BE 53:277.

Custodio, R.; Botanas, C. J.; de la Pena, J. B.; Dela, P. I.; Kim, M.; Sayson, L. V.; Abiero, A.; Ryoo, Z. Y.; Kim, B. N.; Kim, H. J.; Cheong, J. H.(2018).Overexpression of the Thyroid Hormone-Responsive (THRSP) Gene in the Striatum Leads to the Development of Inattentive-like Phenotype in Mice.NEUROSCIENCE 390:141. de la Pena, J. B.; Dela, P. I.; Custodio, R. J.; Botanas, C. J.; Kim, H. J.; Cheong, J. H.(2018).Exploring the Validity of Proposed Transgenic Animal Models of Attention-Deficit Hyperactivity Disorder (ADHD).MOL NEUROBIOL 55:3739.

Drerup, J. M.; Hayashi, K.; Cui, H.; Mettlach, G. L.; Long, M. A.; Marvin, M.; Sun, X.; Goldberg, M. S.; Lutter, M.; Bibb, J. A.(2010).Attention-deficit/hyperactivity phenotype in mice lacking the cyclin-dependent kinase 5 cofactor p35.BIOL PSYCHIAT 68:1163.

Elston, T. W.; Pandian, A.; Smith, G. D.; Holley, A. J.; Gao, N.; Lugo, J. N.(2014).Aniracetam does not alter cognitive and affective behavior in adult C57BL/6J mice.PLOS ONE 9:e104443.

Kim, D.; Yadav, D.; Song, M.(2024).An updated review on animal models to study attention-deficit hyperactivity disorder.TRANSL PSYCHIAT 14:187.

Leffa, D. T.; Panzenhagen, A. C.; Salvi, A. A.; Bau, C.; Pires, G. N.; Torres, I.; Rohde, L. A.; Rovaris, D. L.; Grevet, E. H.(2019).Systematic review and meta-analysis of the behavioral effects of methylphenidate in the spontaneously hypertensive rat model of attention-deficit/hyperactivity disorder.NEUROSCI BIOBEHAV R 100:166.

Leffa, D. T.; Torres, I.; Rohde, L. A.(2018).A Review on the Role of Inflammation in Attention-Deficit/Hyperactivity Disorder.NEUROIMMUNOMODULAT 25:328.

Levin, E. D.; Bushnell, P. J.; Rezvani, A. H.(2011).Attention-modulating effects of cognitive enhancers.PHARMACOL BIOCHEM BE 99:146.

Li, Y.; Yin, A.; Sun, X.; Zhang, M.; Zhang, J.; Wang, P.; Xie, R.; Li, W.; Fan, Z.; Zhu, Y.; Wang, H.; Dong, H.; Wu, S.; Xiong, L.(2017).Deficiency of tumor suppressor NDRG2 leads to attention deficit and hyperactive behavior.J CLIN INVEST 127:4270.

Martin, J. R.; Moreau, J. L.; Jenck, F.(1995).Aniracetam reverses memory impairment in rats.PHARMACOL RES 31:133.

Miller, E. M.; Pomerleau, F.; Huettl, P.; Gerhardt, G. A.; Glaser, P. E.(2014).Aberrant glutamate signaling in the prefrontal cortex and striatum of the spontaneously hypertensive rat model of attention-deficit/hyperactivity disorder.PSYCHOPHARMACOLOGY 231:3019.

Myers, M. M.; Musty, R. E.; Hendley, E. D.(1982).Attenuation of hyperactivity in the spontaneously hypertensive rat by amphetamine.Behav Neural Biol 34:42.

Phillips, H.; Mcdowell, A.; Mielby, B. S.; Tucker, I. G.; Colombo, M.(2019).Aniracetam does not improve working memory in neurologically healthy pigeons.PLOS ONE 14:e0215612.

Regan, S. L.; Williams, M. T.; Vorhees, C. V.(2022).Review of rodent models of attention deficit hyperactivity disorder.NEUROSCI BIOBEHAV R 132:621.

Reisel, D.; Bannerman, D. M.; Schmitt, W. B.; Deacon, R. M.; Flint, J.; Borchardt, T.; Seeburg, P. H.; Rawlins, J. N.(2002).Spatial memory dissociations in mice lacking GluR1.NAT NEUROSCI 5:868.

Reynolds, C. D.; Jefferson, T. S.; Volquardsen, M.; Pandian, A.; Smith, G. D.; Holley, A. J.; Lugo, J. N.(2017).Oral aniracetam treatment in C57BL/6J mice without pre-existing cognitive dysfunction reveals no changes in learning, memory, anxiety or stereotypy.F1000Res 6:1452.

Reynolds, G. D.(2015).Infant visual attention and object recognition.BEHAV BRAIN RES 285:34.

Sagvolden, T.(2006).The alpha-2A adrenoceptor agonist guanfacine improves sustained attention and reduces overactivity and impulsiveness in an animal model of Attention-Deficit/Hyperactivity Disorder (ADHD).BEHAV BRAIN FUNCT 2:41.

Song, Y.; Yuan, H.; Chen, T.; Lu, M.; Lei, S.; Han, X.(2020).An Shen Ding Zhi Ling Alleviates Symptoms of Attention Deficit Hyperactivity Disorder via Anti-Inflammatory Effects in Spontaneous Hypertensive Rats.FRONT PHARMACOL 11:617581.

Togashi, H.; Nakamura, K.; Matsumoto, M.; Ueno, K.; Ohashi, S.; Saito, H.; Yoshioka, M.(2002).Aniracetam enhances glutamatergic transmission in the prefrontal cortex of stroke-prone spontaneously hypertensive rats.NEUROSCI LETT 320:109.

Toriumi, K.; Tanaka, J.; Mamiya, T.; Alkam, T.; Kim, H. C.; Nitta, A.; Nabeshima, T.(2018).Shati/Nat8l knockout mice show behavioral deficits ameliorated by atomoxetine and methylphenidate.BEHAV BRAIN RES 339:207.

Wang, L.; Mu, Z.; Lin, X.; Geng, J.; Xiao, T. Q.; Zhang, Z.; Wang, Y.; Guan, Y.; Yang, G. Y.(2017).Simultaneous Imaging of Cerebrovascular Structure and Function in Hypertensive Rats Using Synchrotron Radiation Angiography.FRONT AGING NEUROSCI 9:359.

Weiss, S.; Nosten-Bertrand, M.; Mcintosh, J. M.; Giros, B.; Martres, M. P.(2007).Nicotine improves cognitive deficits of dopamine transporter knockout mice without long-term tolerance.NEUROPSYCHOPHARMACOL 32:2465.

Wijayawardhane, N.; Shonesy, B. C.; Vaithianathan, T.; Pandiella, N.; Vaglenova, J.; Breese, C. R.; Dityatev, A.; Suppiramaniam, V.(2008).Ameliorating effects of preadolescent aniracetam treatment on prenatal ethanol-induced impairment in AMPA receptor activity.NEUROBIOL DIS 29:81.

Won, H.; Mah, W.; Kim, E.; Kim, J. W.; Hahm, E. K.; Kim, M. H.; Cho, S.; Kim, J.; Jang, H.; Cho, S. C.; Kim, B. N.; Shin, M. S.; Seo, J.; Jeong, J.; Choi, S. Y.; Kim, D.; Kang, C.; Kim, E.(2011).GIT1 is associated with ADHD in humans and ADHD-like behaviors in mice.NAT MED 17:566.

Yuan, H.; Ni, X.; Zheng, M.; Han, X.; Song, Y.; Yu, M.(2019).Effect of catalpol on behavior and neurodevelopment in an ADHD rat model.BIOMED PHARMACOTHER 118:109033.

Zimmermann, A. M.; Jene, T.; Wolf, M.; Gorlich, A.; Gurniak, C. B.; Sassoe-Pognetto, M.; Witke, W.; Friauf, E.; Rust, M. B.(2015).Attention-Deficit/Hyperactivity Disorder-like Phenotype in a Mouse Model with Impaired Actin Dynamics.BIOL PSYCHIAT 78:95.
